# Oral contact dermatitis spicy chewing gum

**DOI:** 10.1186/2045-7022-5-S1-P1

**Published:** 2015-03-11

**Authors:** Gabriel Jose Gonzalez Salazar, Hugo Larrain Paez, Javier Ortiz de Frutos, Sara Lopez, Erika Klar Ferrer, Veronica Monsalvez, Lara Angulo, Francisco Vanaclocha

**Affiliations:** 1 Hospital Universitario doce de Octubre, Madrid, Spain; 2 Doce de Octubre University Hospital, Dermatology, Madrid, Spain; 3 El Escorial University Hospital, Family Medicine, Madrid, Spain; 4 Doce de Octubre University Hospital, pathology, Madrid, Spain

## Background

The oral contact dermatitis caused by cinnamon was initially described by Drake in 1976, in a patient with stomatitis caused by toothpaste containing cinnamon. It is an uncommon mechanisms due to the oral mucosa´s own mechanisms such as saliva that acts as a diluent and buffer, and a low number of antigen presenting cells present. Cases with multiple forms of presentation are described in the literature, the actual incidence is unknown due to the multitude of compounds used mainly in dental prosthetics. The oral contact dermatitis is an uncommon condition caused by various agents. We present the case of a patient with a recurrent glossitis and pain in the oral mucosa, caused by a reaction to the consumption of cinnamon-flavoured chewing gums.

## Case report

A 47 year old woman with no history of interest, attends the emergency room due to tongue pain and erythema with the addition of white adhered lesion that affect the oral mucosa. The patient associated this episode to the chronological intake of cinnamon gum, 2 hours after chewing gum, the lesions appeared.

## Method and results

After a month without lesions, the patient underwent a provocation test with cinnamon flavoured gum and approximately 2 hours later the original lesions began to reappear. In addition, patch tests were performed according to standard GEIDAC battery, battery pastry and bakery and gum as their own, showing positivity for fragrance mix I, balsam of Peru, benzoyl peroxide and to those at 96 and 168 hours. The prick test was negative with the gum.

## Conclusion

Diagnosis is clinical and confirmed by patch tests, which were positive in this patient. The treatment is to avoid the causative agent, in more severe cases corticosteroids ranging from topical to systemic can be used. A case of contact dermatitis from oral consumption of cinnamon gum. We emphasize the importance of a good patient history to reach a proper diagnosis.

## Consent

Written informed consent was obtained from the patient for publication of this abstract and any accompanying images. A copy of the written consent is available for review by the Editor of this journal.

